# Cytotoxicity and Genotoxicity of Panel of Single- and Multiwalled Carbon Nanotubes: *In Vitro* Effects on Normal Syrian Hamster Embryo and Immortalized V79 Hamster Lung Cells

**DOI:** 10.1155/2014/872195

**Published:** 2014-12-08

**Authors:** C. Darne, F. Terzetti, C. Coulais, C. Fontana, S. Binet, L. Gaté, Y. Guichard

**Affiliations:** Département Toxicologie et Biométrologie, Institut National de Recherche et de Sécurité (INRS), rue du Morvan, CS 60027, 54519 Vandoeuvre les Nancy Cedex, France

## Abstract

Carbon nanotubes (CNTs) belong to a specific class of nanomaterials with unique properties. Because of their anticipated use in a wide range of industrial applications, their toxicity is of increasing concern. In order to determine whether specific physicochemical characteristics of CNTs are responsible for their toxicological effects, we investigated the cytotoxic and genotoxic effects of eight CNTs representative of each of the commonly encountered classes: single- SW-, double- DW-, and multiwalled (MW) CNTs, purified and raw. In addition, because most previous studies of CNT toxicity were conducted on immortalized cell lines, we decided to compare results obtained from V79 cells, an established cell line, with results from SHE (Syrian hamster embryo) cells, an easy-to-handle normal cell model. After 24 hours of treatment, MWCNTs were generally found to be more cytotoxic than SW- or DWCNTs. MWCNTs also provoked more genotoxic effects. No correlation could be found between CNT genotoxicity and metal impurities, length, surface area, or induction of cellular oxidative stress, but genotoxicity was seen to increase with CNT width. The toxicity observed for some CNTs leads us to suggest that they might also act by interfering with the cell cycle, but no significant differences were observed between normal and immortalized cells.

## 1. Introduction

Carbon nanotubes (CNTs) belong to the nanomaterials family [1]. Due to their unique specific properties (e.g., size, strength, and electrical conductivity), their use is planned in many industrial areas, including electronics, the medical and pharmaceutical industries, and aeronautics. CNTs make up a complex family, comprising single-walled and multiwalled carbon nanotubes (SWCNTs and MWCNTs) composed of single or multiple graphene sheets rolled into cylinders. CNTs can also be functionalized for industrial purposes through modification of the nanotube surface with specific chemical groups. These surface modifications are generally made for facilitating their integration into composite materials.

The biodurability and high length-to-width aspect ratio of CNTs have raised questions related to their toxicity and effects on human health. Their fibrous nature has led to particular concern surrounding the CNTs, and parallels have been made with asbestos fibres and their effects on humans [2, 3]. To date, occupational exposure to CNTs remains poorly understood, but exposure can occur during their manufacture as well as during their industrial use, for example, in the machining or sanding of carbon parts [4].

During the last decade, many toxicological studies have been published on the potential health effects of CNTs, but the results have been sometimes conflicting. The discrepancy is mainly a result of differences in the type of CNT used (shape, diameter, and being single-walled or multiwalled), the concentrations used, or the dispersion methods employed. Moreover, few studies have analysed SW- and MWCNTs in the same experimental model [5–11].

To illustrate this complexity, CNTs have been shown to induce* in vivo* an inflammatory response after intratracheal instillation [12–17] or intraperitoneal injection with fibrosis and granuloma [2, 13], but the effects were less clear after inhalation [14, 18].* In vitro*, a general decrease in cell viability has been shown [19–21]. Genotoxic events have also been observed* in vitro* with the micronucleus assay [9, 22–24] and the comet assay [22, 24, 25]. In contrast, Asakura et al. [26] observed no induction of micronuclei or hgprt mutations in CHL/IU lung cells, which raises concerns about the relevance of the choice of the cellular type according to nanomaterial and toxicological endpoints. The oxidative stress, induced after treatment with fibers and particles, can explain in part the biological effects observed. For carbon nanotubes, several works have shown that they were able to induce and increase ROS production [27–30].

The main objective of the present study was to determine the toxicological effects of CNTs according to their physicochemical characteristics. However, as the majority of previous studies were conducted on immortalized cell lines and as Syrian hamster embryo cells (SHE) are normal and easily implemented, we also compare the toxicological effects of CNTs on SHE cells and on immortalized Chinese hamster lung fibroblast V79 cells. This comparison will enable us to determine whether a normal cell model is more suitable than an immortalized cell line for evaluating the toxic effects of CNTs.

For this purpose, five commercially available CNTs (one SWCNT, two DWCNTs, and two MWCNTs), which can potentially be found in the workplace, were tested in V79 and SHE cells for their* in vitro* genotoxicity (comet and micronucleus assays), cytotoxicity, and oxidative stress induction (DCFH-DA fluorescent probe). Three other laboratory-synthesized CNTs (one DWCNT and two MWCNTs) were tested for comparison.

## 2. Material and Methods

### 2.1. Samples (Table 1)

The single- and double-walled samples analysed in this study included

- a purified single-walled carbon nanotube (SWCNT 1100, Nanocyl, Belgium);
- a purified double-walled carbon nanotube (DWCNT 2100, Nanocyl, Belgium);
- a short, purified double-walled carbon nanotube (DWCNT 2150, Nanocyl, Belgium) derived from grinding DWCNT 2100;
- a purified double-walled carbon nanotube (DWEF), donated by E. Flahaut of CIRIMAT/UMR CNRS 5085, Toulouse, France [31].

Two multiwalled carbon nanotubes were also tested:

(v) a purified multiwalled carbon nanotube (MWCNT 3100, Nanocyl, Belgium);
(vi) a short, purified multiwalled carbon nanotube (MWCNT 3150, Nanocyl, Belgium), derived from grinding of MWCNT 3100;

Two other MWCNT samples were provided by Dr. D. Begin (LMSPC-UMR 7515-Strasbourg), synthesized according to Gulino et al. [32]:

(vii) a raw multiwalled carbon nanotube (MWCNT SBb);
(viii) a purified multiwalled carbon nanotube (MWCNT SBp).

Several criteria guided our choice of CNT samples. First, five of the samples are commercially available (samples 1100, 2100, 2150, 3100, and 3150) and can therefore be encountered in the workplace. The other three samples (DWEF, SBb, and SBp) were synthesized in research laboratories. Second, each of the large CNT families is represented (single-, double-, and multiwalled CNTs). Third, both short and long CNTs were obtained in order to determine the biological effect of CNT length (2100 versus 2150; 3100 versus 3150). Finally, both raw and purified samples were chosen in order to determine the impact of the presence of chemical products other than carbon on cellular toxicity (2100 versus DWEF; SBb versus SBp).

### 2.2. Physicochemical Characterisation of Samples (Table 1)

The chemical contents of CNT samples were analysed by inductively coupled plasma mass spectrometry (ICP-MS) (Spectro Ciros CCD, Germany). Nanotube diameters and the number of walls present were measured by transmission electron microscopy (TEM) (Philips CM20, The Netherlands). Specific surface area was determined using the BET technique [33] with a Gas Sorption Analyzer (ASAP 2020 Micromeritics, France). CNT lengths were determined by the supplier.

### 2.3. Sample Preparation and Cell Treatments

In order to obtain a homogeneous suspension (estimated visually), the samples were placed in complete medium at the highest concentration used in* in vitro* assays and sonicated for 2.5 min. with a VibraCell (50 W, 20 KHz, Bioblock Scientific, France) at 40% power.

DLS (dynamic light scattering) analysis was done to determine the agglomeration status of suspensions using a Zetasizer Nano ZS apparatus (Malvern, France), but as mentioned before by Tavares et al. [34] and as recently commented on by the OECD [35], such technique (designed for analysis of spherical particles) did not give correct results (data not shown). The alternative method, electronic microscopy, involves methods of sample preparation which induce changes in agglomeration status and thus is not perfectly adapted either. In the absence of adequate technique, the agglomeration status was unknown.

The SHE and V79 cells were treated with CNTs at concentrations ranging from 0.27 to 2.1 *μ*g/cm^2^ of cell culture dish (free radical generation) or from 0.23 to 3.75 *μ*g/cm^2^ (other assays). These concentrations are in the same range as those previously used in our laboratory for studies of asbestos fibres [36]. In a preliminary experiment, these concentrations induced no more than 50–55% cytotoxicity as measured by the WST assay (see below).

### 2.4. Cell Culture

V79 cells (lung fibroblast from Chinese hamster, ATCC, USA, reference CCL-93) were selected for this study as they are one of the cell models recommended in OCDE guideline number 487 for use in the* in vitro* micronucleus assay. Cells were grown in Dulbecco's MEM (DMEM; Invitrogen, France), supplemented with 10% fetal calf serum (Dutscher, France) and 0.5% Penicillin/Streptomycin (5000 U-5000 *μ*g/mL, Invitrogen, France). Cells were incubated at 37°C with 10% CO_2_, as recommended by the supplier for optimal culture with our medium.

Syrian hamster embryo (SHE) cell cultures were used as they are normal diploid cells, nongenetically modified, metabolically competent, and p53 effective and there is no known difference with those constituting the organism where they come from. They have been demonstrated to be suitable for genotoxicity assays [37, 38]. Cells were established from individual 13-day gestation foetuses (inbred colony, INRS, France). The culture medium used was Dulbecco's MEM (DMEM; Invitrogen, France), supplemented with 17% fetal calf serum (Dutscher, France) and 0.5% Penicillin/Streptomycin (5000 U-5000 *μ*g/mL, Invitrogen, France). Cells were incubated at 37°C and 10% CO_2_.

### 2.5. Cell Viability

1 × 10^3^ cells/mL (V79) or 1.5 × 10^3^ cells/mL (SHE) were seeded in 48 wells on a 96-well plate for 24 h. The cell cultures were then treated for 24 h with culture medium (control) or with sample suspensions in final concentrations between 0.23 and 3.75 *μ*g/cm^2^ of cell culture surface. The remaining 48 wells on the well plate received the same suspensions (medium or CNT samples) to ensure the absence of interference between CNTs and WST-1 reagent. After treatment, 1/10 (v/v) WST-1 reagent (Roche Diagnostics, France) was added to each well for 3 h. The plates were then centrifuged at 4500 rpm for 5 min to eliminate the majority of CNTs and therefore to avoid interference between the soluble formazan dye formed and the CNTs at the time of the reading. The supernatant was transferred to new 96-well plates and optical density (OD) was recorded at 450 nm and 690 nm with a microtiter plate reader (Synergy HT, BioTek, France,). The delta OD (OD450 nm–OD 690 nm) was then calculated. At least three independent experiments were realized for every point.

Data were expressed as % of control ± SEM for each treatment concentration and compared using an ANOVA-LSD test (Fisher's least significant difference) (Statgraphics Centurion, Statpoint Technologies, USA).

Cell counting for the comet assay was performed with a Coulter Z1 (Beckman Coulter, France) (data not shown).

### 2.6. Free Radical Generation

5 × 10^4^ V79 or SHE cells were treated with 0.27 to 2.1 *μ*g/cm^2^ of CNTs for 24 h. Thirty minutes before the end of treatment, 25 *μ*M of 2′,7′-dichlorodihydrofluorescin diacetate (H_2_DCF-DA, Invitrogen, France) was added to the cultures. Cells were trypsinized and then centrifuged and then placed in HBSS (Hank's buffer saline solution) with 50 *μ*g/mL of propidium iodide. Fluorescence was measured using a Becton Dickinson FACStar^PLUS^ flow cytometer. A sample of nanometric anatase TiO_2_ [37] was used for the positive control at 9.2 *μ*g/cm^2^. Two independent experiments (with duplicates) were realized for every point. Data were expressed as the mean fluorescence intensity of the two experiments ± SD. Statistical analysis was performed using an ANOVA-LSD test (Fisher's least significant difference) (Statgraphics Centurion, Statpoint Technologies, USA).

Potential interference between CNTs and DCF was tested by acellular assays, mixing H_2_DCF (obtained by NaOH treatment of H_2_DCF-DA) or DCF fluorescent probe (Sigma-Aldrich, France) and CNTs at different concentrations (from 1 to 250 *μ*g/mL, equivalent to 0.23 to 58 *μ*g/cm^2^ of cell culture dish). No interference was shown for up to 25 *μ*g/mL (5.8 *μ*g/cm^2^) of CNTs in SHE cells (data not shown).

### 2.7. Comet Assay

The Fpg modified comet assay was used to evaluate oxidative DNA damage. The Fpg enzyme, a glycosylase, recognizes and specifically cuts modified bases such as 8-oxoguanine from DNA, producing apurinic sites that are converted into strand breaks by the associated AP-endonuclease activity. Therefore, DNA strand breaks detected by the Fpg modified comet assay provide a measure of oxidative DNA damage [39]. We followed the procedure of Collins et al. [40], with minor modifications.

In brief, two duplicate comet slides were made for each treatment: one slide was treated with Fpg and the other with the Fpg buffer only. The two slides were subsequently treated in the same manner.

The SHE (2 × 10^5^) or V79 (1 × 10^5^) cells were treated for 24 hours either with CNTs at concentrations ranging from 0.23 to 3.75 *μ*g/cm^2^ or with positive control methyl methanesulfonate (MMS, Sigma-Aldrich, France) at 0.125 mM or with medium alone.

Approximately 20,000 cells were mixed in 600 *μ*L of 1% low melting agarose (LMA, Sigma-Aldrich, France) and the mixture was transferred onto a slide precoated with normal melting agarose (NMA 1%, Sigma-Aldrich, France). Slides were then immersed in lysis solution (2.5 M NaCl, 100 mM Na_2_EDTA, and 10 mM Tris with 1% Triton X-100 and 10% DMSO added fresh) and kept in the dark for 1 h at 4°C.

The slides were drained and incubated in the dark for 30 min at 37°C, either in enzyme buffer alone or in Fpg (5 U/mL) in enzyme buffer (40 mM HEPES, 0.1 M KCl, and 0.5 mM Na_2_EDTA; pH 8). The slides were immersed in cold alkaline solution (300 mM NaOH, 1 mM Na_2_EDTA; pH 13) for 20 min and electrophoresis was then performed in the same buffer at 0.7 V/cm for 40 min to allow the fragments of damaged DNA to migrate towards the anode. The slides were then washed with 0.4 M Tris-HCl for 15 min and stained with propidium iodide (2.5 *μ*g/mL).

Slides were examined at 200x magnification under a fluorescence microscope. Images of 100 randomly selected comets were acquired and analyzed for each sample (comet assay IV, Perceptive Instruments, UK) in order to evaluate the % tail DNA used as a measure of DNA damage. The presence of CNTs did not interfere with the reading at the concentrations tested. The experiment was repeated three times independently. Statistical analyses were performed on means using the ANOVA-LSD test (Statgraphics Centurion, Statpoint Technologies, USA). The *P* < 0.05 level was considered to be statistically significant. The concentration/tail DNA relationship was determined by linear regression (mixed model) after logarithmic transformation of tail DNA and concentration values (STATA 12.1, College Station, Texas, USA).

### 2.8. Micronucleus Test

Approximately 2.5 × 10^4^ V79 cells and 5 × 10^4^ SHE cells were seeded in Labtek slides (Nunc A/S, Denmark) with 1 mL of culture medium. After 24 h, the cells were treated either with CNTs at concentrations ranging from 0.23 to 3.75 *μ*g/cm^2^ or with positive control methyl methanesulfonate (MMS, Sigma-Aldrich, France) at 0.25 mM or with medium alone for 24 h (V79 cell doubling time: 14–18 hours; SHE cell doubling time: 18–20 hours). At the end of treatment, cells were washed with PBS (phosphate buffer saline, Invitrogen, France) and fixed in methanol for 15 min. Slides were washed in PBS and drained and received one drop of Pro Long Gold antifade reagent with DAPI (Molecular Probe, Invitrogen, France). About 1000 cells were analysed at each concentration for the presence of micronuclei (MN). The presence of CNTs did not interfere with the reading. Each assay was repeated three times. Cell proliferation/division was assessed through analysis of the mitotic index (% of mitotic cells). Statistical analysis of MN induction was performed on the pooled data of the three independent experiments using the Chi-square test. The *P* < 0.05 level was considered to be statistically significant.

## 3. Results

### 3.1. Physicochemical Characterisation of Samples (Table 1)

The single-walled 1100 CNT sample contained 3.15 wt. % silica and 1.44 wt. % cobalt. The double-walled 2100 and 2150 CNT samples contained 2.69 and 2.48 wt. % molybdenum and 1.79 and 1.4 wt. % iron, respectively. The double-walled DWEF CNT sample contained 9.5 wt. % cobalt. The multiwalled 3100, 3150, and SBp samples contained few impurities, but the MWCNT SBb contained 7.22 wt. % aluminium and 4.15 wt. % iron. The TEM analyses revealed that most of the metal catalysts were located inside the carbon nanotubes. Specific surface areas were higher for single- (1128 m^2^/g) and double-walled CNT (611 to 985 m^2^/g) than for the multiwalled CNT (between 150 and 330 m^2^/g). Due to the association of carbon nanotubes in bundles, it was not possible to accurately measure their lengths. The external diameters of the carbon nanotube samples ranked from small to large as follows: 1100 (1.5–4 nm) < DWEF (1.6–3.4 nm) < 2100–2150 (3–7 nm) < 3100–3150 (11–19 nm) < SBb-SBp (9–77 nm).

After dispersion in complete medium, optical microscopy observations showed that the MWCNTs were better dispersed than both the double-walled and single-walled CNT (1100), even though bundles were present in all samples.

### 3.2. ROS Generation

**Figure 1:**
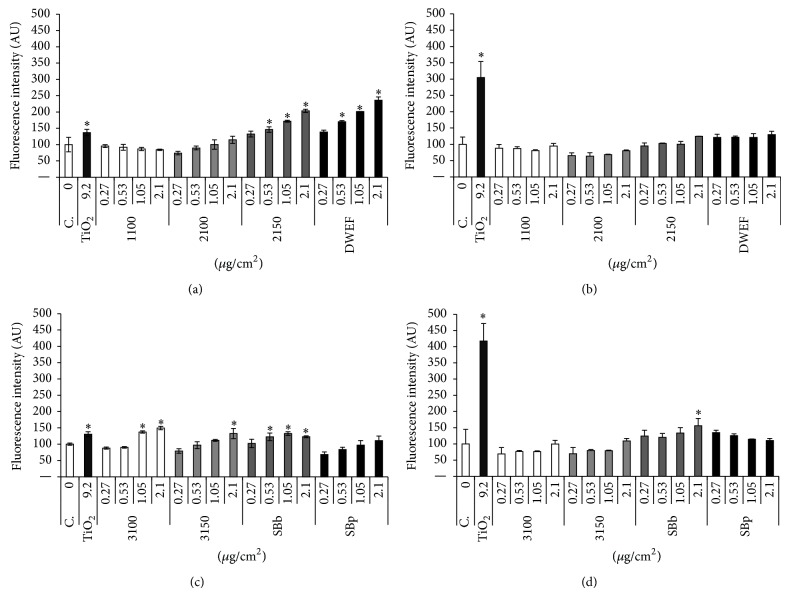
Oxidative stress after 24 h of treatment with CNTs, expressed as fluorescence intensity (% of control ± SD) in V79 and SHE cells. Fluorescence intensity with single- (1100) or double-walled carbon nanotubes (2100–2150, DWEF) in (a) V79 cells and (b) SHE cells and with multiwalled carbon nanotubes (3100–3150, SBb and SBp) in (c) V79 cells and (d) SHE cells. C.: control (medium alone); TiO_2_: positive control. Two independent experiments with duplicate were realized for every point. Data were expressed as the mean fluorescence intensity of the two independent experiments ± SD. Sample concentrations are expressed as *μ*g per cm^2^ of cell culture surface. ^*^ Statistically significant (*P* < 0.05) compared to control.

The production of reactive oxygen species (ROS) is often associated with toxicological effects of particles or fibres. In order to address this issue, we performed ROS detection in cells after treatment with CNTs, using the cell-permeable DCFH-DA fluorogenic probe. As shown in Figures 1(a) and 1(b), no increase in fluorescence intensity was induced by exposure of either cell type to the 1100 single-walled carbon nanotubes or by the 2100 double-walled carbon nanotubes. The 2150 and DWEF samples induced significant increases in fluorescence with concentration in V79 cells (Figure 1(a)) but not in SHE cells (Figure 1(b)). For the MWCNTs in V79 cells, only the SBp sample did not induce significant increase in fluorescence. The 3150 induced significant increase at the highest dose, the 3100 at the two highest concentrations (1.05 and 2.1 *μ*g/cm^2^), and SBb at 0.53, 1.05, and 2.1 *μ*g/cm^2^. In SHE cells, all MWCNT samples were negative except sample SBb, which induced a significant increase at the highest concentration (2.1 *μ*g/cm^2^) (Figures 1(c) and 1(d)).

### 3.3. Cell Viability

**Figure 2:**
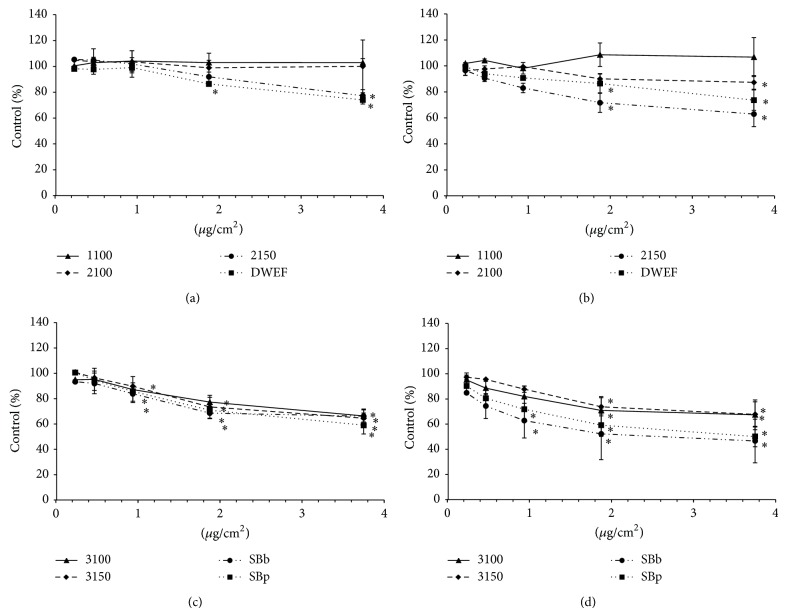
Effect of carbon nanotubes on cell viability assessed by the WST assay. Results are expressed as the percentage of delta OD (OD 450 nm–OD 690 nm) in treated cells ± SD compared to control cells (100 %) after 24 h of treatment with CNT samples. Single- or double-walled carbon nanotubes in (a) V79 cells and (b) SHE cells. Multiwalled carbon nanotubes in (c) V79 cells and (d) SHE cells. Sample concentrations are expressed as *μ*g per cm^2^ of cell culture surface. At least three independent experiments were realized for every point. ^*^ Statistically significant (*P* < 0.05) decrease in cell viability compared to control.

Cell viability was assessed after 24 h treatment with the carbon nanotube samples (Figure 2). The data are reported as the percentage of control relative to the concentration. 24-hour exposure to the SWCNT 1100 sample caused no modification of cell viability in either cell type, regardless of the concentration tested (0.23 to 3.75 *μ*g/cm^2^). The 2100 DWCNT induced a significant reduction in cell viability at 3.75 *μ*g/cm^2^ in SHE cells but not in V79 cells. All the other SW- and DW-carbon nanotubes induced a concentration-dependent decrease in cell viability, which became significant at 3.75 *μ*g/cm^2^ in V79 cells and at 1.87 *μ*g/cm^2^ in SHE cells for the 2150 sample and at 1.88 and 3.75 *μ*g/cm^2^ in both cell types for the DWEF sample. All the MWCNTs induced significant decreases in cell viability at the two (3100 in V79 and SHE; 3150 in SHE) or three (3150, SBb, and SBp) highest concentrations. The effect on cell viability of the samples at 3.75 *μ*g/cm^2^ ranked in the following order:

 in V79 cells: 1100–2100 (100–102% of control) < 2150 (77%) < DWEF (74%) < 3100-SBb (66%) < 3150 (64%) < SBp (59%);
 in SHE cells: 1100 (106%) < 2100 (87%) < DWEF (74%) < 3100–3150 (67%) < 2150 (63%) < SBp (50%) < SBb (47%).

In conclusion, the MWCNTs were found to be more cytotoxic than both the SW- or DW-nanotubes.

### 3.4. Genotoxicity

Two types of assay were used to evaluate the genotoxicity of carbon nanotubes in V79 and SHE cells: the comet assay and the micronucleus assay.

#### 3.4.1. Comet Assay

**Figure 3:**
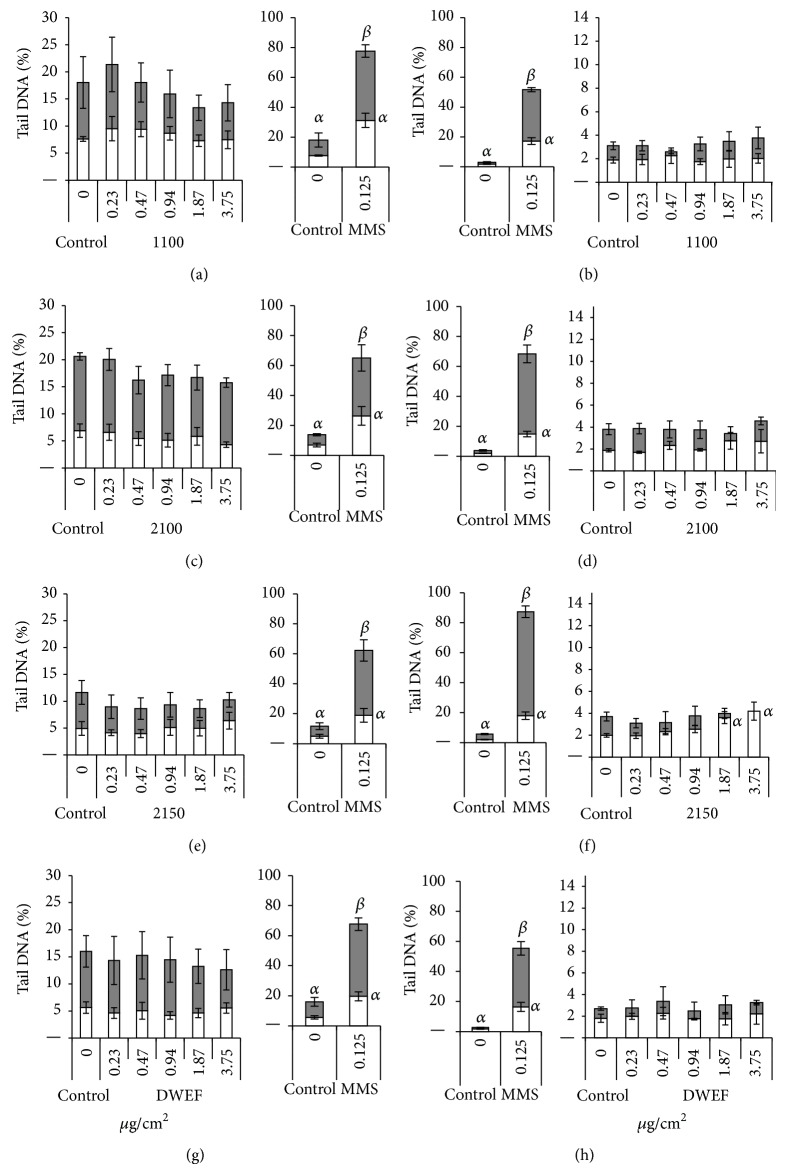
DNA damage in cells after 24-hour treatment with SW- and DWCNTs, expressed as tail DNA (%) ± SEM. For each CNT, the small histogram represents the results obtained with the negative (medium) and positive (0.125 mM MMS) control, both with (filled histogram) and without (open histogram) the Fpg enzyme. The large histogram represents data obtained for different concentrations of CNT. Sample concentrations are expressed as *μ*g per cm^2^ of cell culture surface. Three independent experiments were realized for each point. *α*: statistically significant (*P* < 0.05) compared to control; *β*: statistically significant (*P* < 0.05) compared to Fpg control. Data obtained for the negative control are shown on both histograms (note the different scales). The significance mark was omitted from the small histogram for better reading of the graph. (a), (c), (e), and (g) DNA breaks in V79 cells. (b), (d), (f), and (h) DNA breaks in SHE cells.

Results obtained following 24-hour treatment with 1100, 2100, 2150, and DWEF samples are presented in Figure 3. The positive control (MMS) induced significant DNA damage in both V79 and SHE cells, both with and without the Fpg enzyme treatment. For the negative control (medium alone), an increase in the number of DNA breaks was observed after treatment of the slides with the Fpg enzyme.

With the exception of sample 2150, treatment of the cells with the SW- or DWCNT samples induced no effect in either V79 or SHE cells, with or without Fpg treatment. Sample 2150 induced a significant increase in the number of DNA breaks at 1.87 and 3.75 *μ*g/cm^2^ in the absence of Fpg in SHE cells (Figure 3(f)).

**Figure 4:**
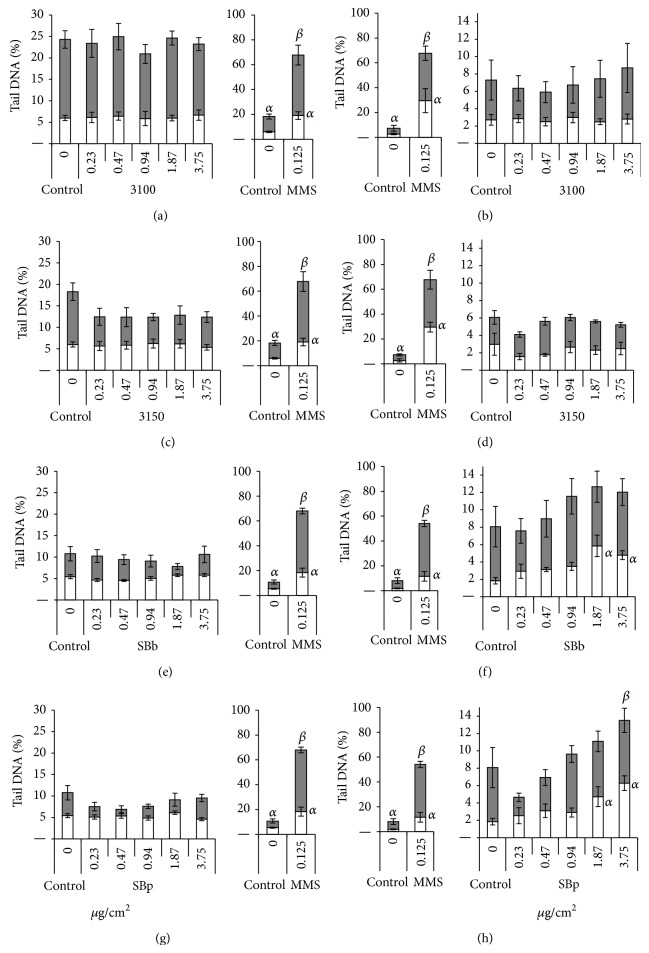
DNA damage in cells after 24-hour treatment with MWCNTs, expressed as tail DNA (%) ± SEM. For each CNT, the small histogram represents the results obtained with negative (medium) and positive (0.125 mM MMS) control, both with (filled histogram) and without (open histogram) the Fpg enzyme. The large histogram represents data obtained for different concentrations of CNT. Sample concentrations are expressed as *μ*g per cm^2^ of cell culture surface. Three independent experiments were realized for each point. *α*: statistically significant (*P* < 0.05) compared to control; *β*: statistically significant (*P* < 0.05) compared to Fpg control. Data obtained for the negative control are shown on both histograms (note the different scales); the significance mark was omitted from the small histogram for better reading of the graph. (a), (c), (e), and (g) DNA breaks in V79 cells. (b), (d), (f), and (h) DNA breaks in SHE cells.

Negative results were also obtained in V79 cells with MWCNTs (Figure 4), regardless of the concentration tested and both with and without Fpg treatment. In SHE cells, only the SBb and SBp samples showed a significant concentration-damage relationship, with a significant increase in DNA breaks observed at the two highest concentrations (Figures 4(f) and 4(h)). We also observed a concentration-related increase in damage after Fpg treatment, with a significant response at the highest concentration for the SBp sample (Figure 4(h)).

#### 3.4.2. Micronucleus Assay (Table 2)

In V79 cells, sample 1100 induced a significant increase in micronucleated cells at concentrations of 0.94 and 1.87 *μ*g/cm^2^ but not at 3.75 *μ*g/cm^2^. The results obtained from exposure to the double-walled CNTs (2100, 2150, and DWEF) also showed one (2150: 0.23 *μ*g/cm^2^) or two (2100: 0.23, 0.94 *μ*g/cm^2^; DWEF: 0.47, 0.94 *μ*g/cm^2^) significant concentrations. In SHE cells in contrast, samples 1100, 2100, and 2150 had no effect, and DWEF only induced a significant increase in the number of micronucleated cells at 0.23 *μ*g/cm^2^.

The 3100 MWCNT induced a significant increase in the number of micronucleated cells at concentrations of 0.23, 0.47, and 1.87 *μ*g/cm^2^ in V79 cells and at 0.47 *μ*g/cm^2^ in SHE cells.

The 3150 MWCNT exhibited similar genotoxic potential in that three (0.23, 0.94, and 1.87 *μ*g/cm^2^) and four (0.23, 0.94, 1.87, and 3.75 *μ*g/cm^2^) of the concentrations tested induced significant increases in the number of micronucleated cells in SHE and V79 cell cultures, respectively. Micronucleus formation was significant in V79 cells at all concentrations for SBb and SBp samples, with a concentration relationship observed for the SBb sample. The SBb and SBp samples were also positive in SHE cells but only at three concentrations (0.23, 0.47, and 0.94 *μ*g/cm^2^) for the SBp sample and four concentrations (0.23, 0.47, 0.94, and 1.87 *μ*g/cm^2^) for the SBb sample.

The V79 mitotic index shows that all CNTs with the exception of the 1100 and DWEF samples induce a decrease in the number of cells in mitosis. This effect was more pronounced for the MWCNTs than for the single- or double-walled CNTs and correlates with cell viability if sample DWEF is excluded (Figure 2). This certainly corresponds to a cessation of cell division. In SHE cells, only SBb and SBp, and to a lesser extent 2150, induced a decrease in the mitotic index.

## 4. Discussion

The specific physicochemical properties of carbon nanotubes, associated with their high aspect ratios, have led many laboratories to initiate and conduct* in vitro* and* in vivo* toxicological studies. However, results are sometimes conflicting and despite these efforts it is difficult to draw any overall conclusions. In this work, eight CNTs representative of each of the commonly encountered classes (single- (SW-), double- (DW-), and multiwalled (MW) CNTs, purified and raw) were tested for their cytotoxicity and genotoxicity in SHE and V79 cells. V79 cells, which are recommended for the micronucleus assay (OECD guideline number 487), have also been used for comet assays in several studies. SHE cells were used as they are primary cells and are suitable for analyzing the genotoxic properties of chemicals and in particular the effects of fibres or particles [36–38].

### 4.1. Discussion of Results

We have shown that, in our experimental conditions, MWCNTs were more cytotoxic than their single- or double-walled equivalents in both cell types. SHE cells and V79 cells do not present any great differences in terms of sensitivity. Because the SB samples induced 50–55% cytotoxicity at concentrations of 3.75 *μ*g/cm^2^, higher concentrations would not have been compatible with the other assays for evaluating the genotoxic potential of CNTs.

Even though comparison with other studies is complex and risky because of differences between the materials and methods used, we note that our results differ from those obtained after 24-hour treatment in both macrophage NR8383 [7] and human aortic endothelial cells [41] in which the same toxicity was observed for SW- and MWCNTs at concentrations of 31.2 *μ*g/cm^2^ and 1.4 *μ*g/cm^2^, respectively. Several hypotheses can be put forward to explain the discrepancy between these results. First, the different methods used for sample dispersion may have caused some discrepancy in biological assays as the CNTs may have been dispersed to different extent. Second, some CNTs may interfere with the culture medium, leading to cytotoxicity through nutrient depletion [42, 43]. We tested this hypothesis in a preliminary experiment by incubating the culture medium with CNTs. After CNT elimination, no SHE cell cytotoxicity or cytostasis was induced by the medium (data not shown). A third hypothesis invokes metal catalyst particles as an actor of cytotoxicity [7]. However, we observed only slight differences between the SBb and SBp samples. Moreover, electronic microscopy showed that the metal particles are located inside the CNTs and therefore do appear to be in contact with the surrounding medium. Sample 3150 is the only sample that has also been used in other studies. Interestingly, as in our study, Chen et al. [30] observed that the 3150 sample induced cytotoxicity in A549 human lung epithelial cells (34%) and in RAW 264.7 murine macrophage cells (27%) but at much higher concentrations (25 *μ*g/mL, approximately 15 *μ*g/cm^2^) than we observed.

Cell number decrease, as measured by the WST viability test, and the decrease in cell mitosis for the majority of samples in both cell types, as measured by the mitotic index, suggest that CNTs can act on the cell cycle and block cell division, as was observed in C6 rat glioma cells with MWCNTs [44]. This phenomenon could therefore be part of the cytotoxicity. One possible explanation for this could be that the action of CNTs mainly takes place at the level of mitotic spindle as was demonstrated in the studies of Sargent et al. [45–47]. The action of CNTs on the cell cycle should therefore be investigated in a future study, by analysis of the cell cycle, the DNA repair system, DNA synthesis, and the spindle apparatus.

MWCNTs, on the whole, also had greater effects than SW- and DWCNTs in the genotoxicity assays. However, unlike in the cytotoxicity assays, some differences were observed in the responses of the two cell types. In the comet assay, none of the CNTs induced a significant increase in DNA damage in V79 cells, whereas SBb and SBp (MWCNTs) and 2150 (DWCNT) induced significant increases in the number of DNA breaks at the two highest concentrations in SHE cells. Treatment with the Fpg enzyme increased the level of DNA breakage in both cell types (see control assays with and without Fpg in Figures 3 and 4), indicating that there was a background level of DNA base modification in cells. However, no significant difference was observed between control Fpg and treated Fpg cells in V79, and in SHE cells, only SBp triggered a significant increase in damage at 3.75 *μ*g/cm^2^ compared to the Fpg treated control. Similar results were obtained with the Fpg enzyme by Cavallo et al. [48] in their investigation of MWCNT genotoxicity in A549 cells.

An increase in the number of DNA breaks induced by the Fpg enzyme is often associated with the presence of oxidized bases. Oxidative stress and production of reactive oxygen species are described as cytotoxic and genotoxic effectors which can lead to the production of oxidized bases. This was demonstrated in different cellular types with the cell-permeable DCFH-DA fluorogenic probe after treatment with SWCNTs [27] or MWCNTs [30, 49, 50]. In our case, even though we were able to observe significant ROS production with the DCFH-DA probe for some CNTs, no clear relationship could be identified between ROS production and DNA damage. Similarly, no link could be made between ROS production and cytotoxicity. The effects of CNT-induced ROS production should be investigated in more detail by examining the levels of superoxide dismutase and glutathione and by using ROS scavengers.

CNT samples were also shown in our study to be capable of inducing micronucleated cells in both cell types, and the effect was seen to be more pronounced with MWCNTs. The most genotoxic CNTs were the 3150 MWCNT, whose length is described as short by the supplier, the raw and the purified SB samples. The decrease in micronucleated cell frequency at the highest concentrations may be explained by a cell cycle arrest, as was also suggested by the decrease in the mitotic index (see the previous section). Our results from the micronucleus assay corroborate those obtained by others with both SW- and MWCNTs [9, 20]. For example, Migliore et al. [9], who demonstrated that SW- and MWCNTs induce the formation of micronuclei in RAW 264 cells, also showed that these CNTs can induce DNA damage. The same results were obtained by Pacurari et al. [25, 51] in human mesothelial cells.

To summarize, our results show that some CNTs, and mainly the MWCNTs, can induce cytotoxicity and genotoxicity in SHE and V79 cells. Furthermore, because the CNTs induced more micronucleated cells than DNA damage and as CNT exposure provoked a cell cycle arrest as revealed by the evaluation of the mitotic index, we can hypothesize that CNTs may act on the apparatus spindle during cell division.

When looking at the* in vivo* studies for a comparison and even if such exercise is limited in terms of conclusions, the MWCNTs seem to be more genotoxic than SWCNTs as we have shown in the present study. But,* in vivo,* data are limited and results obtained for the SWCNTs present some discrepancies. Genotoxic effects have been seen in mouse or rat with SWCNTs by some authors [52–54] but not by others [55–58]. For MWCNTs, results are less confusing with a majority of studies showing genotoxic effects [59–61]. One study has compared SW- and MWCNTs in the same model with the same methodology but in this work both SW- and MWCNTs were unable to induce genotoxic effects [57, 62].

### 4.2. Comparison of V79 and SHE Cells

A number of comments can be made regarding the responses of the two cellular types, taking into account that CNTs are present in both cellular types as early as 3 h of treatment (electronic microscopy analysis, data not shown): (i) CNT cytotoxicity is at almost the same level in both cell types; (ii) more ROS were generated in V79 cells than in SHE cells exposed to CNTs; (iii) more micronucleated cells were observed after CNT treatment in V79 cells, but no DNA damage was revealed by the comet assay; the opposite of that was observed in SHE cells for SBb and SBp.

V79 cells are immortalized cells. As they can undergo an infinite number of cell divisions and even though no genotyping or metabolism data were available for this clone, the enzymatic content and gene expression profile for V79 cells are most probably modified at the level of cell cycle checkpoints and DNA repair pathways. These differences can explain both the higher level of ROS production compared to SHE cells and the higher background of DNA breaks observed in control V79 compared to normal SHE cells. However, the p53 protein, which mediates the cellular response to DNA damage and is involved in cell cycle regulation, apoptosis, and DNA repair [63], does not appear to be able to explain these differences. Indeed, V79 cells have already been described as defective for the functional p53 protein. As shown by Chaung et al. [64], the V79 p53 sequence contains two mutation points that result in a nonfunctional protein [64]. Conversely, SHE cells are normal diploid cells with no alterations in the cell cycle pathway [65, 66] and SHE cells also contain a normal p53 protein [67, 68]. However, even if V79 cells do have a mutated p53 gene, we showed in the present study that CNTs induced the same cytotoxicity and induced micronucleated cell formation in both cell types. These findings corroborate those of Hashimoto et al. [69], who found no difference in sensitivity to micronucleus induction and cytotoxicity in p53-wild and p53-null human lymphoblastoid cells. Furthermore, the mitotic index suggests that a cell cycle arrest occurs in both cell types following exposure to CNTs. Thus, this blockage does not appear to be influenced by the presence or absence of a mutated p53 gene.

To examine this further, as we suggested earlier, additional experiments should be conducted to investigate the cell cycle, spindle apparatus, and effectiveness of the DNA repair system. An analysis, at the mRNA and protein levels, of p53 and mdm2 (E3 ubiquitin ligase that inactivates p53 by binding directly) in SHE cells, could be also beneficial to better understanding of the response of these cells.

As mentioned before, our results show that, for a given CNT, the ROS generation can be different according to the cell type. In acellular assay, we have shown that all CNTS were able to induce DCF fluorescence in phosphate buffer up to 25 mg/mL (corresponding to 5.8 *μ*g/cm^2^) (data not shown). As the basal level of ROS was the same in terms of fluorescence intensity in both cell types and as CNTs were able to induce ROS in acellular assay, the level of ROS cell generation seems to be specific to a combination between CNT and cellular type. These are preliminary results and ROS production should be investigated in more detail by examining the levels of enzyme content of each cellular type, the response to ROS scavengers, and so on.

However, our results nevertheless suggest that no large differences exist between the V79 cell line and the SHE normal cells after CNT treatment. The two cellular types are thus complementary and a benefit can certainly be gained in using SHE cells as they are normal cells that are appropriate for the evaluation of nanomaterial cytotoxicity and genotoxicity.

### 4.3. CNT Characteristics and Biological Effects

Regarding the physicochemical properties and biological effects of CNTs, the most pronounced cytotoxic and genotoxic effects were obtained with the multiwalled SBb and SBp samples, and the least toxic CNTs in our experiments were the SW- and DWCNTs.

Our data also demonstrate that, in our experimental conditions, there is no relationship between the toxicological effects of CNTs and their metal contaminants. Indeed, SBb, which contains 7.22% aluminium and 4.15% iron, presented near-identical toxicological effects to SBp, which contains only 0.86% iron. Concerning surface area, our results suggest that increased toxicity is not correlated with a higher specific surface area. However, it is important to note that the BET method uses a gas to determine the surface area, and therefore the value obtained does not reflect the real surface area in contact with a liquid or biomolecules. Furthermore, the agglomeration status of the suspension used, which we were unable to determine in this study, could directly influence the biological response.

The biological impact of CNT length is also unclear from our experiments. Even though the “shorter” 2150 sample was found to be more cytotoxic and induced more ROS than the “longer” 2100 sample, the two samples exhibited near-identical genotoxic effects. The “long” 3100 and “short” 3150 samples also presented no differences.* In vivo*, Muller et al. [13] found that ground CNTs were less toxic than unground CNTs but concluded that the agglomeration state of the CNTs rather than their length was likely to be responsible for these differences. The same conclusion was reached by Sato et al. [70] in their* in vitro *and* in vivo *studies. However in a later study, Han et al. (2012) [44] observed a more toxic effect from short CNTs than from long CNTs, concluding that CNT length was indeed responsible for the observed difference in toxicity in C6 rat glioma cells.

In our study, the only physical parameters that we were able to partially link to toxic effects were the number of walls and the outer diameters of the CNTs. Certainly, the thickest CNT samples (SBb and SBp) produced the most toxic effects (in terms of both cytotoxicity and genotoxicity). The importance of CNT diameter as a parameter to be considered in toxicology assessment has previously been suggested in the work of Fenoglio et al. [71]. Using two MWCNTs of the same length range but with very different diameters, they showed that the thickest CNT was the least toxic in a murine macrophage cell line (MH-S).

In conclusion, this* in vitro *study demonstrates that exposure to some but not all CNTs induces cytotoxic and genotoxic effects, to different extent depending on the cell type used. Our results also suggest that some CNTs may act on the cell cycle and on cellular division without having any genotoxic effect.

Because of their different physicochemical properties, CNTs have different toxicological profiles. This suggests that it is not possible to draw any general conclusions regarding the toxicity of these nanomaterials.

## Figures and Tables

**Table 1:** Physicochemical characteristics of carbon nanotube samples.

Name	Type	Carbon purity (%)^2^	Amorphous carbon^1,2^	Nb. of walls^1^	Ext. diameter (nm)^1^	Ext. diameter (nm)^2^	Length (*µ*m)^2^	SSA (m^2^/g)^3^	Chemical content (% of mass)^4^
1100	SW purified	>70	Yes	1-2	1.5–4	2	>1	1128	3.15Si; 1.44Co; 0.14Mg
2100	DW purified	>90	Yes	<5	3–7	3.5	1–10	626	2.69Mo; 1.79Fe; 0.16Si; 0.11Ca
2150	DW short purified	>90	Yes	<5	3–7	3.5	>1	611	2.48Mo; 1.40Fe; 0.10Si; 0.12Ca
DWEF	DW purified (80% DW, 15% SW, and 5% TW)	~90,5	n.d.	2	1.6–3.4	1–3	1–20	985	9.5Co^2^
3100	MW purified	>95	n.d.	4-5	11-12	9.5	1.5	333	0.22Fe; 0.1Co
3150	MW short purified	>95	n.d.	4-5	15–19	9.5	<1	308	0.21Fe
SBb	MW raw	>88	<2%	4–10	15–68	15–50	>0.8	151	7.22Al; 4.15Fe
SBp	MW purified	>98	n.d.	4–10	9–77	15–50	>0.8	168	0.86Fe

^1^TEM analysis (INRS).

^2^Manufacturer data.

^3^BET analysis.

^4^ICP-MS analysis (Ag, AL, As, B, Ba, Be, Bi, Ca, Cd, Ce, Co, Cr, Cu, Fe, K, La, Li, Mg, Mn, Mo, Ni, Pb, Sb, Se, Si, Sn, Sr, Ti, V, Zn, and U).

**Table 2:** Induction of micronucleated cells after 24 h of treatment with CNTs in V79 and SHE cells.

Chemical	Concentration (*μ*g/cm^2^)	% of cells with MN		Mitotic index (%)	
		V79 cells	SHE cells	V79 cells	SHE cells
Control	0	1.8	5.1	6.1	1.5

MMS	0.25 mM	18.5^*^	14.1^*^	3.5^*^	2.6^*^

1100	0.23	2,2	4,9	4,8	1,8
	0.47	2,2	5,3	5,1	1,9
	0.94	2,6^*^	4,5	5,8	1,7
	1.87	2,7^*^	3,7	7,5	2,0
	3.75	2,1	4,8	5,4	1,9

2100	0.23	2,6^*^	5,3	4,4^*^	1,7
	0.47	2,3	5,6	5,1	1,9
	0.94	2,7^*^	5,1	5,0	1,9
	1.87	1,9	5,3	4,3^*^	1,4
	3.75	1,9	4,7	4,4^*^	1,8

2150	0.23	2,6^*^	5,7	6,4	1,9
	0.47	2,2	5,9	4,4^*^	1,9
	0.94	1,6	4,9	4,4	1,3
	1.87	2,0	4,7	5,0	0,8^*^
	3.75	1,5	4,1	4,7^*^	1,3

DWEF	0.23	2,2	6,8^*^	5,8	1,5
	0.47	2,7^*^	5,9	5,0	1,7
	0.94	2,5^*^	5,6	6,1	1,2
	1.87	2,0	4,7	5,5	1,3
	3.75	1,8	4,7	6,1	1,1

3100	0.23	2,9^*^	6,3	4,7^*^	1,6
	0.47	3,0^*^	6,8^*^	4,6^*^	1,4
	0.94	2,4	6,2	2,9^*^	2,0
	1.87	2,5^*^	5,5	3,0^*^	1,2
	3.75	1,5	5,4	3,1^*^	1,2

3150	0.23	2,6^*^	7,7^*^	4,4	1,4
	0.47	2,0	7,1^*^	3,0^*^	1,6
	0.94	2,9^*^	6,4^*^	3,2^*^	1,3
	1.87	2,5^*^	5,8	3,1^*^	1,2
	3.75	3,2^*^	5,9	3,6^*^	1,2

SBb	0.23	3,1^*^	6,6^*^	5,1	1,9
	0.47	3,6^*^	6,7^*^	5,1	0,9^*^
	0.94	4,6^*^	6,5^*^	4,3^*^	0,8^*^
	1.87	5,5^*^	6,4^*^	4,4^*^	0,3^*^
	3.75	5,6^*^	4,4	3,5^*^	0,2^*^

SBp	0.23	3,8^*^	8,0^*^	6,1	1,1
	0.47	3,4^*^	6,7^*^	4,6^*^	1,0
	0.94	3,8^*^	6,9^*^	4,8	0,8^*^
	1.87	3,7^*^	5,6	5,0	0,7^*^
	3.75	2,7^*^	3,4	2,8^*^	0,2^*^

Data presented were established with at least 3000 cells derived from three independent assays. ^*^Statistically significant (*P* < 0.05).
